# Patterns and Driving Mechanisms of β‐Diversity in Mountain Plant Communities of Arid Regions

**DOI:** 10.1002/ece3.72886

**Published:** 2026-01-12

**Authors:** Tiantian Qin, Hongyang Chen, Shengjie Chen, Pengwei Zhang, Zhifang Xue, Shengtianzi Dong, Hanyue Wang

**Affiliations:** ^1^ College of Life Sciences Shihezi University Shihezi China; ^2^ Key Laboratory of Xinjiang Phytomedicine Resource Utilization, Ministry of Education Shihezi University Shihezi China; ^3^ Xinjiang Production and Construction Corps Key Laboratory of Oasis Town and Mountain‐Basin System Ecology Shihezi University Shihezi China; ^4^ Urumqi Center for Comprehensive Survey of Natural Resources China Geological Survey Urumqi China

**Keywords:** community assembly mechanisms, northern slope of the central Kunlun Mountains, phylogenetic β‐diversity, taxonomic β‐diversity, β‐diversity components

## Abstract

Arid mountain ecosystems are characterized by unique environmental stresses and high biodiversity. However, knowledge regarding the distribution patterns, multidimensional characteristics, and shaping forces of plant community β‐diversity—especially under extreme environmental conditions—remains insufficient. In this study, we focused on the northern slope of the central Kunlun Mountains, an extremely arid region, and analyzed plant community survey data from 72 sites. By applying β‐diversity partitioning and multiple regression analyses, we systematically identified the patterns of both taxonomic and phylogenetic β‐diversity and their components and evaluated the relative importance of geographic, climatic, and topographic distances in shaping β‐diversity. The results showed that species turnover is the primary force structuring both taxonomic and phylogenetic β‐diversity, and that these diversity indices increase substantially with elevational differences. The combined influence of geographic, climatic, and topographic distances explained 46.4% of the variability in taxonomic β‐diversity and 53.6% in phylogenetic β‐diversity. Variance partitioning analysis showed that β‐diversity patterns were shaped predominantly by climatic distance, while the contributions of geographic and topographic distances were limited. Among climatic variables, mean annual temperature emerged as the most influential determinant. This study not only deepens our understanding of biodiversity maintenance mechanisms under extreme environmental conditions but also provides important scientific evidence for the formulation of biodiversity conservation and management strategies in this region, particularly for optimizing the spatial layout of multiple small nature reserves under a turnover‐dominated β‐diversity pattern.

## Graphical abstract

We analyzed taxonomic and phylogenetic β‐diversity patterns in plant communities along the northern slope of the central Kunlun Mountains in arid northwestern China. Species turnover dominated β‐diversity, with climatic distance—particularly temperature—being key drivers. These results emphasize the role of environmental filtering under extreme arid conditions.

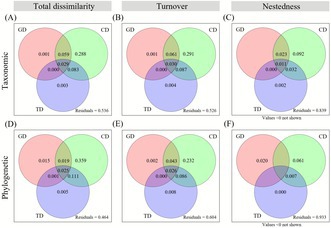

## 1 Introduction

Mountain ecosystems, with their steep environmental gradients and pronounced habitat heterogeneity, have long been regarded as natural laboratories for exploring the mechanisms underlying biodiversity and community assembly (Körner et al. 2017; Muellner‐Riehl et al. 2019). Among these, arid mountain ecosystems merit special attention because they combine the dual constraints of elevation and water scarcity, exhibiting sharp temperature fluctuations, limited moisture, and strong climatic seasonality (Magaña Ugarte et al. 2019; Ma et al. 2024). Unlike humid montane systems such as the Andes or Himalayas—where high energy and water availability favor dense vegetation and biotic interactions—arid mountains are stress‐dominated environments in which abiotic filtering plays a central role in shaping community composition (Xu et al. 2019; Zheng et al. 2022). These systems therefore provide an exceptional setting to evaluate how environmental filtering and dispersal limitation interact under extreme climatic conditions, offering a critical test of general biodiversity theories such as the neutral model (Hubbell 2001) and the water‐energy dynamics hypothesis (Hawkins et al. 2003). Understanding these processes is increasingly urgent, as ongoing aridification and human disturbances across Central Asia threaten the stability and resilience of these fragile mountain ecosystems (Mori et al. 2018; Rahbek et al. 2019).

β‐diversity, which quantifies variation in species assemblages across communities, is widely recognized as a key ecological metric for investigating the mechanisms governing community assembly and biodiversity maintenance (Whittaker 1960; Socolar et al. 2016). Traditional taxonomic β‐diversity emphasizes species‐level differences but fails to comprehensively reflect the evolutionary history and phylogenetic relationships within communities (Graham and Fine 2008; Du et al. 2021). More recently, incorporating phylogenetic β‐diversity has introduced new theoretical perspectives for disentangling the impacts of niche differentiation, neutral processes, and lineage diversification during community assembly (García‐Girón et al. 2020; Wang et al. 2022). Based on the partitioning framework, β‐diversity can be divided into two principal components: turnover and nestedness, each corresponding to different ecological drivers such as environmental filtering, limits to dispersal, and ordered extinction or colonization events (Baselga 2012; Qian and Ricklefs 2012; Bishop et al. 2015). Turnover describes shifts in assemblage structure resulting from the substitution of species along ecological or spatial gradients, whereas nestedness represents the role of ordered species loss or gain in shaping regional diversity patterns (Legendre 2014; Fontana et al. 2020). Thus, adopting a multidimensional perspective on β‐diversity allows for a deeper interpretation of the biotic mechanisms governing both community formation and diversity patterns (Zheng et al. 2022; Li et al. 2024).

Prior investigations have indicated that the distribution of β‐diversity in plant communities is largely shaped by the combined impacts of geographic distance (serving as an indicator of dispersal limitation) and environmental variables (reflecting the action of environmental filters), with climatic and topographic factors being especially influential (Bohlman et al. 2008; Liang et al. 2025). However, the relative strength of these mechanisms differs greatly across ecosystems. In humid and temperate systems, such as those in China and North America, β‐diversity is more strongly linked to environmental distance (Qian et al. 2013, 2020; Yang et al. 2022), whereas in African and arid grassland ecosystems, dispersal limitation tends to dominate community differentiation (Li et al. 2021; Qian et al. 2024). Increasing evidence suggests that both mechanisms usually operate simultaneously, with their balance influenced by climate, habitat heterogeneity, and evolutionary history (Myers et al. 2013; Bellier et al. 2014; Hu et al. 2022). Despite global progress, little is known about how these processes jointly regulate β‐diversity and its turnover and nestedness components in extremely arid mountain ecosystems, where both climatic stress and geographic isolation are pronounced. Addressing this uncertainty is essential for understanding the mechanisms underlying biodiversity maintenance in Central Asian drylands.

The northern slope of the central Kunlun Mountains forms a typical mountain‐basin system in Central Asian drylands, acting as a key ecological transition and biogeographic convergence zone between the Kunlun Mountains and the Tarim Basin (Li 1960; Cui et al. 1988). Controlled by continental arid circulation and the uplift of the Qinghai–Tibet Plateau, this region features sharp gradients in temperature, precipitation, and elevation, supporting a continuous vegetation spectrum from desert steppe to alpine meadow (Ma et al. 2025). Owing to its strong environmental heterogeneity and long‐term geological and climatic dynamics, the area functions as an important refugium and differentiation center for dryland flora, preserving relict lineages and facilitating species migration across the plateau–basin interface (Gui et al. 2010; Du et al. 2022). These attributes make the Kunlun Mountains an ideal natural laboratory for testing β‐diversity hypotheses, where the combined effects of water limitation, topographic complexity, and spatial isolation create distinct assembly processes along elevation. However, despite increasing floristic and vegetation surveys, comprehensive analyses integrating taxonomic, phylogenetic, environmental, and spatial data remain lacking in this unique arid‐mountain system.

Given the unique geographic and climatic setting of the northern slope of the central Kunlun Mountains and the limited understanding of how its extreme environment shapes community differentiation, this study investigates how turnover and nestedness collectively structure taxonomic and phylogenetic β‐diversity along elevation, and how geographic, climatic, and topographic distances drive their variation. By integrating these dimensions, the study aims to clarify the relative roles of environmental filtering and dispersal limitation in arid mountain ecosystems, thereby advancing community assembly theory and providing a scientific basis for biodiversity conservation in Central Asian drylands.

## 2 Materials and Methods

### 2.1 Research Area and Vegetation Sampling

**FIGURE 1:**
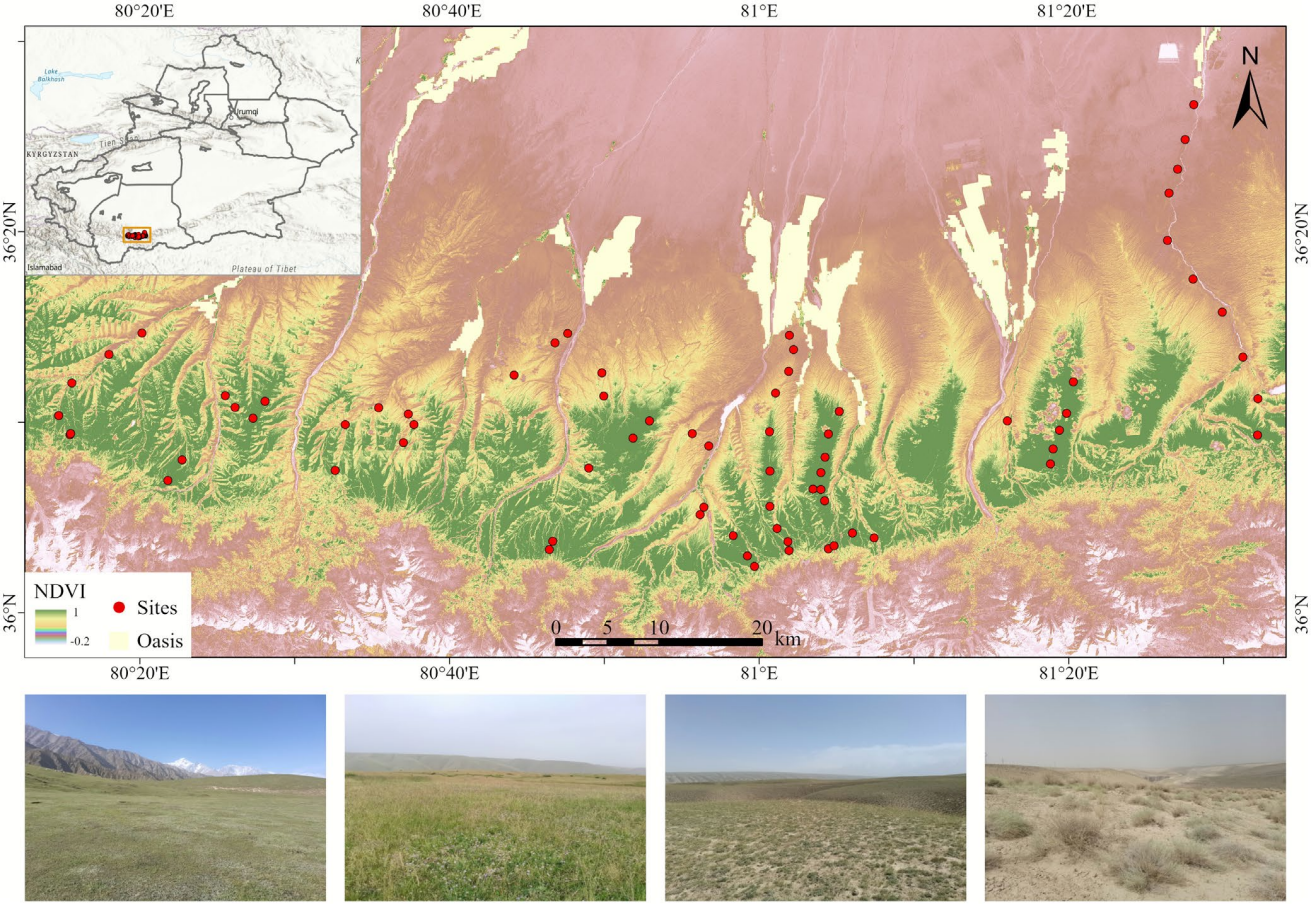
Study area and sampling plot distribution on the northern slope of the central Kunlun Mountains (photo credits: the authors).

The northern slope of the central Kunlun Mountains lies under the influence of the Mongolian–Siberian dry anticyclone and exhibits a distinctly continental climate—characterized by pronounced aridity, low precipitation, warm summers, and cold winters. Proximity to the Taklimakan Desert further exacerbates the degree of drought in the region. Consequently, dryness in this area far exceeds that of the Altai range as well as the northern flanks of the Tianshan, with drought especially intense in the lower mountain zones (Cui et al. 1988). The hydrothermal conditions vary markedly across different elevation zones of the Kunlun Mountains, with temperature decreasing progressively with increasing altitude. Steppe is the dominant vegetation type on the northern slope of the central Kunlun Mountains, where plant species richness is relatively low, reflecting the region's arid and cold climatic conditions (Gui et al. 2010). The study area (36°02′36″‐36°26′46″ N, 80°14′38″‐81°32′19″ E; Figure 1) experiences a mean annual temperature of 7.7°C (January: −7.86°C; July: 16.23°C), with an annual accumulated temperature above 10°C reaching 2715°C. Mean annual precipitation is only 33 mm (Ma et al. 2025), about 70% of the total precipitation falls within the primary period of plant growth, which occurs from May to July each year. Across the elevational gradient, plant communities display distinct zonation, encompassing a range of vegetation types, including montane desert, montane desert steppe, typical montane steppe, and alpine steppe (Cui et al. 1988). The dominant soils are desert brown soils and calcic brown soils.

Field investigations were conducted from June to August 2023, when plant growth was at its peak. Considering site accessibility, we established 72 sampling sites across the study area, stratified by altitudinal gradients and vegetation types, at elevations ranging from 1950 to 3690 m. At each site, one 20 × 20 m plot was randomly placed in areas with relatively homogeneous community composition, structure, and habitat conditions. Within each plot, five 5 × 5 m shrub quadrats were established at the four corners and the center, and a 1 × 1 m herbaceous quadrat was nested within each shrub quadrat (Zheng et al. 2022). Following the community survey protocol of Fang et al. (2009), we recorded all plant species, their abundance, cover, and mean height within each quadrat. To fully capture species composition at each site, we additionally recorded species present in the main plot but absent from the nested quadrats. For each 20 × 20 m plot, the geographic coordinates of the plot center and its four corners were recorded using a GPS device to provide spatial references for subsequent extraction of DEM‐derived topographic variables.

### 2.2 Environmental Data

We selected seven climatic variables representing the key thermal and hydrological dimensions that strongly influence vegetation patterns in arid mountain ecosystems (Wang et al. 2012; Qian et al. 2020). These variables include mean annual temperature (MAT), temperature seasonality (TS), mean annual precipitation (MAP), precipitation seasonality (PS), mean temperature of the warmest month (MTWM), mean temperature of the coldest month (MTCM), and potential evapotranspiration (PET). All climatic data were obtained from the National Tibetan Plateau Data Center (TPDC, https://data.tpdc.ac.cn/), which provides a 1‐km resolution dataset derived from the WorldClim and CRU climate databases through a delta downscaling approach validated by 496 meteorological stations.

The selection of these seven variables was based on their ecological relevance to plant growth and community assembly in arid mountain regions, representing the major climatic dimensions of thermal energy (MAT, MTWM, MTCM), water availability (MAP, PET), and seasonal variability (TS, PS). Rather than including the full set of bioclimatic variables, we focused on a limited number of widely used and ecologically interpretable predictors that have been shown to capture the principal climatic gradients in dryland and alpine ecosystems. This approach helps to reduce redundancy among highly correlated climate variables while maintaining clear ecological meaning (Qian and Ricklefs 2012; Zheng et al. 2022; Wen et al. 2024).

Topographic variables, including elevation, slope, aspect, and topographic wetness index (TWI), were extracted from the ALOS 12.5 m resolution digital elevation model (DEM; https://www.resdc.cn/) using ArcGIS Pro 3.0.2. Although elevation was recorded in the field, DEM‐based extraction was necessary to obtain fine‐scale and spatially consistent estimates of terrain attributes (slope, aspect, and TWI) that cannot be accurately measured in situ across all plots. These variables were subsequently used, together with climatic factors, to characterize environmental heterogeneity in the statistical analyses.

### 2.3 Phylogenetic Tree Reconstruction

The phylogenetic tree was constructed using “V.PhyloMaker”, following the APG IV system for family and genus structure (Byng et al. 2016; Jin and Qian 2019), after standardizing all species names with the “plantlist” package (Zhang et al. 2022). For species not included in the backbone tree, the recommended “Scenario 3” approach was adopted, attaching missing taxa to their corresponding genus or family nodes.

### 2.4 Computation and Partitioning of β‐Diversity Indices

To differentiate taxonomic (TD) and phylogenetic (PD) β‐diversity within plant communities, we applied three pairwise dissimilarity metrics implemented in the “betapart” R package (Baselga 2010; Baselga and Orme 2012). The Sørensen index (βsor) was employed to represent total β‐diversity. Its partitioning yields the Simpson index (βsim), quantifying the turnover fraction, and the nestedness index (βnes), which isolates the component attributable to species richness differences among communities.

All analyses were conducted using presence/absence data, following the formulation of the betapart framework. Although species abundance and cover were recorded in the field, we adopted the presence/absence approach to ensure methodological consistency between taxonomic and phylogenetic β‐diversity metrics. Moreover, abundance‐based indices such as Bray‐Curtis were not applied because cover estimates may vary with observer bias and microhabitat heterogeneity across mountain plots, potentially obscuring large‐scale compositional patterns.

### 2.5 Data Analysis

In this study, geographic, climatic, and topographic distances among the 72 sampling plots were quantified using the Euclidean distance metric. Spatial coordinates (latitude and longitude) of each sampling location were used to compute pairwise geographic distances, following established procedures (Tan et al. 2013; Wang et al. 2013). Because community similarity typically declines exponentially with greater distance, a logarithmic (log_10_) transformation was applied to the geographic distance matrix to better capture distance‐decay relationships (Ruokolainen and Tuomisto 2002).

To address potential collinearity among environmental variables and to facilitate dimensionality reduction, we performed principal component analysis (PCA) independently for both climatic and topographic datasets prior to all further analyses. For climate factors, the first two principal components (PC1 and PC2) accounted for 83.9% and 13.9% of the total variance, respectively, thus providing an efficient summary of the seven original climatic factors. The first three principal components of the topographic variables explained 37.5%, 24.2%, and 20.6% of the variance, respectively, accounting for 82.3% of the total variation and effectively capturing the major gradients among the four topographic factors.

Statistical comparisons of β‐diversity between the two focal dimensions were performed using two‐sided Wilcoxon rank‐sum tests (Tan et al. 2013). Relationships between β‐diversity metrics (including turnover and nestedness) and the three categories of environmental distances were assessed with Mantel tests, implemented using Spearman's rank correlation coefficient (*r*_*s*_) as the association measure (Smouse et al. 1986). Correlation strengths were classified as strong (|*r*_*s*_| > 0.66), moderate (0.33 < |*r*_*s*_| ≤ 0.66), or weak (|*r*_*s*_| ≤ 0.33), in line with previous ecological studies (Qian et al. 2020). Although the Mantel test has been criticized for potential pseudoreplication and inflated significance in spatial analyses, it remains an appropriate and widely applied method for evaluating correlations between ecological and environmental dissimilarity matrices (Legendre et al. 2015). In this study, Mantel tests were used to assess the overall correspondence between β‐diversity and environmental distances, consistent with the test's intended scope.

To disentangle the respective influences of climatic and topographic distances (environmental filtering) and geographic distance (dispersal limitation) on β‐diversity, multiple regression on distance matrices (MRM) was conducted (Li et al. 2024). The first MRM analysis served as the basis for variance partitioning analysis (VPA), quantifying the relative contributions of geographic, climatic, and topographic distances to β‐diversity patterns (Wu et al. 2019; Li et al. 2024). Adjusted *R* ^2^ values were used to correct for differences in predictor numbers and avoid overestimation of explained variation. Negative fractions, which lack biological interpretation, were not displayed in the figures. They usually occur when using adjusted *R* ^2^, indicating weak explanatory power or collinearity among variables, but were retained in supplementary tables (Table S2) for transparency.

In addition, a second MRM was performed to identify which specific environmental variables exerted the strongest influence on β‐diversity and its components. Prior to this analysis, multicollinearity among the 11 initial environmental variables (7 climatic and 4 topographic) was examined using pairwise Pearson correlation and variance inflation factor (VIF) analyses. Variables showing strong correlations (|*r*| > 0.7) or VIF > 5 were excluded. Highly correlated climatic variables (e.g., TS, PS, MTCM, MTWM, PET) and elevation were removed to avoid redundancy, while five representative variables with low collinearity and clear ecological significance—mean annual temperature (MAT), mean annual precipitation (MAP), slope, aspect, and topographic wetness index (TWI)—were retained for modeling. All *p*‐values were obtained through permutation tests (Legendre et al. 1994).

Prior to analysis, all environmental distance matrices were standardized in R using the “scale” function to eliminate the effects of differing units. Geographic distances were calculated with the “geosphere” package (Hijmans 2021). Mantel tests, multiple regression on distance matrices (MRM), and variance partitioning analysis (VPA) were all conducted in the R 4.2.2 environment (R Core Team 2022), primarily utilizing the “vegan” (Oksanen et al. 2019) and “ecodist” (Goslee and Urban 2007) packages.

## 3 Results

### 3.1 Floristic Composition

A total of 91 plant species belonging to 63 genera and 25 families were recorded across all plots (Table S1), including five small shrubs, 25 annual herbs, and 61 perennial herbs. The dominant species were *Potentilla multifida* L. (Rosaceae), *Leontopodium nanum* (Hook. f. & Thomson ex C. B. Clarke) Hand.‐Mazz. (Asteraceae), *Elymus nutans* Griseb. (Poaceae), *Seriphidium rhodanthum* (Rupr.) Poljakov (Asteraceae), *Astragalus tibetanus* Benth. ex Bunge (Fabaceae), *Stipa purpurea* Griseb. (Poaceae), *Festuca kryloviana* Reverd. (Poaceae), and *Krascheninnikovia ceratoides* (L.) Gueldenst. (Amaranthaceae), among others.

### 3.2 Component Characteristics of Taxonomic and Phylogenetic β‐Diversity and Their Variation With Elevational Differences

**FIGURE 2:**
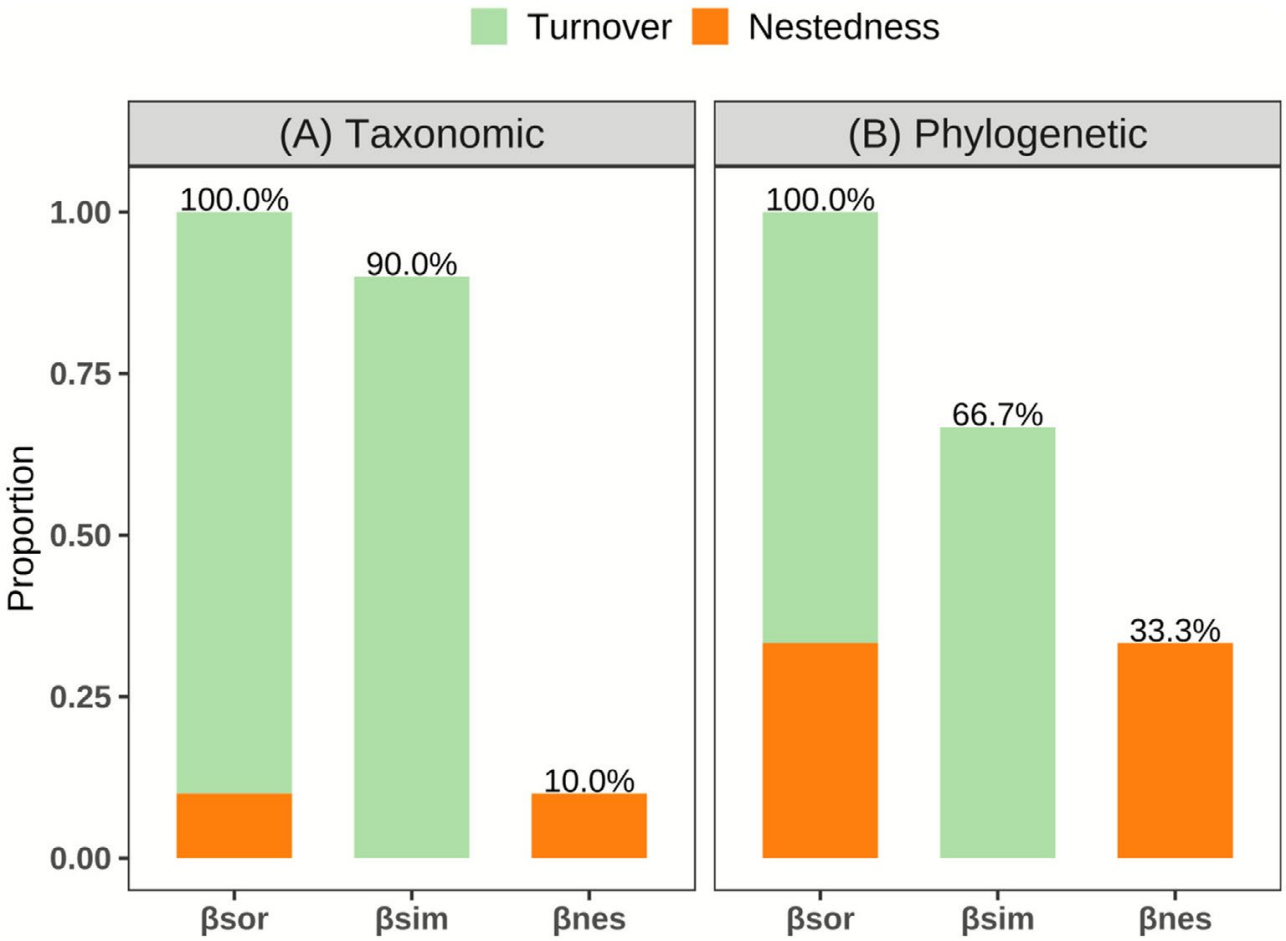
Relative contributions of species turnover and nestedness to total β‐diversity in mountain plant communities across two diversity dimensions. (A) Taxonomic; (B) Phylogenetic. Colored segments represent the proportion of turnover (green) and nestedness (orange) components, with percentages indicating their respective contributions.

**FIGURE 3:**
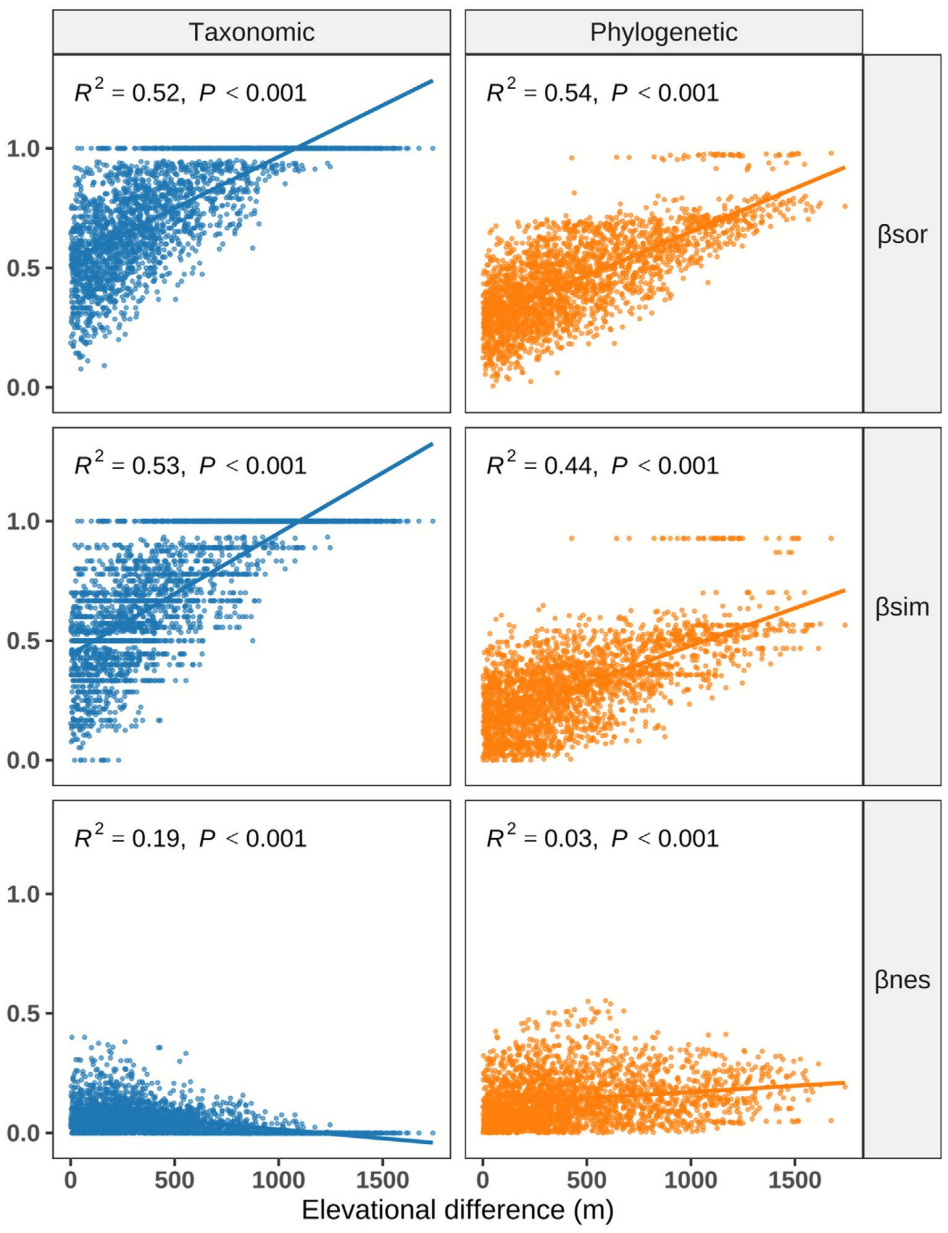
Variation trends of taxonomic and phylogenetic β‐diversity and their components along elevational differences in plant communities. Solid dots represent observed values, and solid lines indicate fitted relationships. *R* ^2^ represents the adjusted coefficient of determination (adjusted *R* ^2^), and each panel also shows the corresponding *p*‐values. The increasing trends of β‐diversity and turnover with elevation indicate intensified species and lineage replacement driven by environmental gradients, whereas the weaker response of nestedness reflects limited effects of ordered species loss.

The results of β‐diversity partitioning (Figure 2) indicated that, in the taxonomic dimension, overall β‐diversity was predominantly driven by species turnover (accounting for 90.0%), with nestedness contributing a much smaller proportion (10.0%). In the phylogenetic dimension, turnover likewise constituted the main component (66.7%), while the contribution of nestedness was 33.3%. The relatively low contribution of nestedness is also ecologically meaningful, suggesting that community differentiation is mainly driven by species replacement rather than by ordered species loss, reflecting high spatial turnover across heterogeneous habitats. There was a strong and significant correlation between βsor and βsim for both taxonomic and phylogenetic dimensions (*r*_*s*_ > 0.66, *p* < 0.001), whereas the correlation involving βnes was notably weaker (*r*_*s*_ = 0.206, *p* < 0.001; Table 1). In addition, both taxonomic and phylogenetic β‐diversity in mountain plant communities increased significantly with greater elevational differences (Figure 3, *p* < 0.001). In both dimensions, overall β‐diversity (βsor) and turnover (βsim) showed a strong positive correlation with elevational differences, and both demonstrated high goodness‐of‐fit, while the correlation between nestedness (βnes) and elevation was relatively weak (Figure 3).

**TABLE 1:** Mantel correlations between taxonomic and phylogenetic β‐diversity (βsor) and its turnover (βsim) and nestedness (βnes) components in plant communities.

Correlation	Component	*r*_*s*_	*p*
Taxonomic/Phylogenetic	βsor	0.8751	0.001
	βsim	0.7629	0.001
	βnes	0.2061	0.001

*Note:* The *r*_*s*_ values represent Spearman's rank correlation coefficients.

### 3.3 Effects of Geographic, Climatic, and Topographic Distances on β‐Diversity and Its Components

#### 3.3.1 Relationships Between β‐Diversity and Spatial‐Environmental Distances

In mountain plant communities, both taxonomic and phylogenetic β‐diversity, along with their turnover and nestedness components, showed significant correlations with geographic, climatic, and topographic distances (Table 2). Among these predictors, climatic distance exhibited the strongest association with total β‐diversity (Tβsor, Pβsor) and their turnover components (Tβsim, Pβsim), with *rₛ* values ranging from 0.587 to 0.706 (*p* < 0.001), whereas correlations with the nestedness components (Tβnes, Pβnes) were weaker or nonsignificant. This pattern suggests that climatic heterogeneity exerts the strongest control over community differentiation, promoting species and lineage replacement across environmental gradients, while nestedness reflects more localized compositional differences.

**TABLE 2:** Mantel correlations between taxonomic and phylogenetic β‐diversity of mountain plant communities and geographic, climatic, and topographic distances.

Index	Tβsor	Tβsim	Tβnes	Pβsor	Pβsim	Pβnes
Geographic D	0.236***	0.245***	−0.210 ns	0.185***	0.228***	−0.037 ns
Climatic D	0.706***	0.702***	−0.464 ns	0.690***	0.587***	0.263***
Topographic D	0.385***	0.384***	−0.286 ns	0.393***	0.359***	0.115*

*Note:* The *r*_*s*_ values correspond to Spearman's rank correlation coefficients.

Abbreviation: ns, not significant.

*** *p* < 0.001.

* *p* < 0.05.

#### 3.3.2 Combined Effects of Geographic, Climatic, and Topographic Distances

Results from the MRM (Table 3) showed that the joint model including geographic, climatic, and topographic distances provided the best overall fit. The combined predictors accounted for 46.4% of the variation in taxonomic β‐diversity and 53.6% in phylogenetic β‐diversity. They also explained a substantially higher proportion of variation in turnover (47.4% for taxonomic and 39.6% for phylogenetic) than in nestedness (16.1% and 6.7%, respectively), indicating that β‐diversity variation is mainly driven by the combined effects of spatial, climatic, and topographic factors, with a more pronounced influence on species replacement (turnover) than on species loss (nestedness).

**TABLE 3:** Results of multiple regression on distance matrices (MRM) for taxonomic and phylogenetic β‐diversity (Tβsor, Pβsor) and their turnover (Tβsim, Pβsim) and nestedness (Tβnes, Pβnes) components under various variable combinations.

Models	Tβsor	Tβsim	Tβnes	Pβsor	Pβsim	Pβnes
GD + CD + TD	0.464***	0.474***	0.161***	0.536***	0.396***	0.067**
GD + CD	0.461***	0.471***	0.159***	0.530***	0.388***	0.067***
GD + TD	0.176***	0.183***	0.069***	0.177***	0.165***	0.007 ns
CD + TD	0.462***	0.473***	0.161***	0.521***	0.394***	0.048**
GD	0.090***	0.093***	0.034***	0.061***	0.071***	0 ns
CD	0.459***	0.469***	0.159***	0.514***	0.386***	0.048***
TD	0.115***	0.121***	0.046***	0.142***	0.120***	0.006 ns

*Note:* GD, CD, and TD denote geographic, climatic, and topographic distances, respectively. The seven models were used in the variance partitioning analysis presented in Figure 4. *R* ^2^ represents the adjusted coefficient of determination (adjusted *R* ^2^), indicating the proportion of β‐diversity variation explained by each model after accounting for model complexity.

Abbreviation: ns, not significant.

*** *p* < 0.001.

** *p* < 0.01.

**FIGURE 4:**
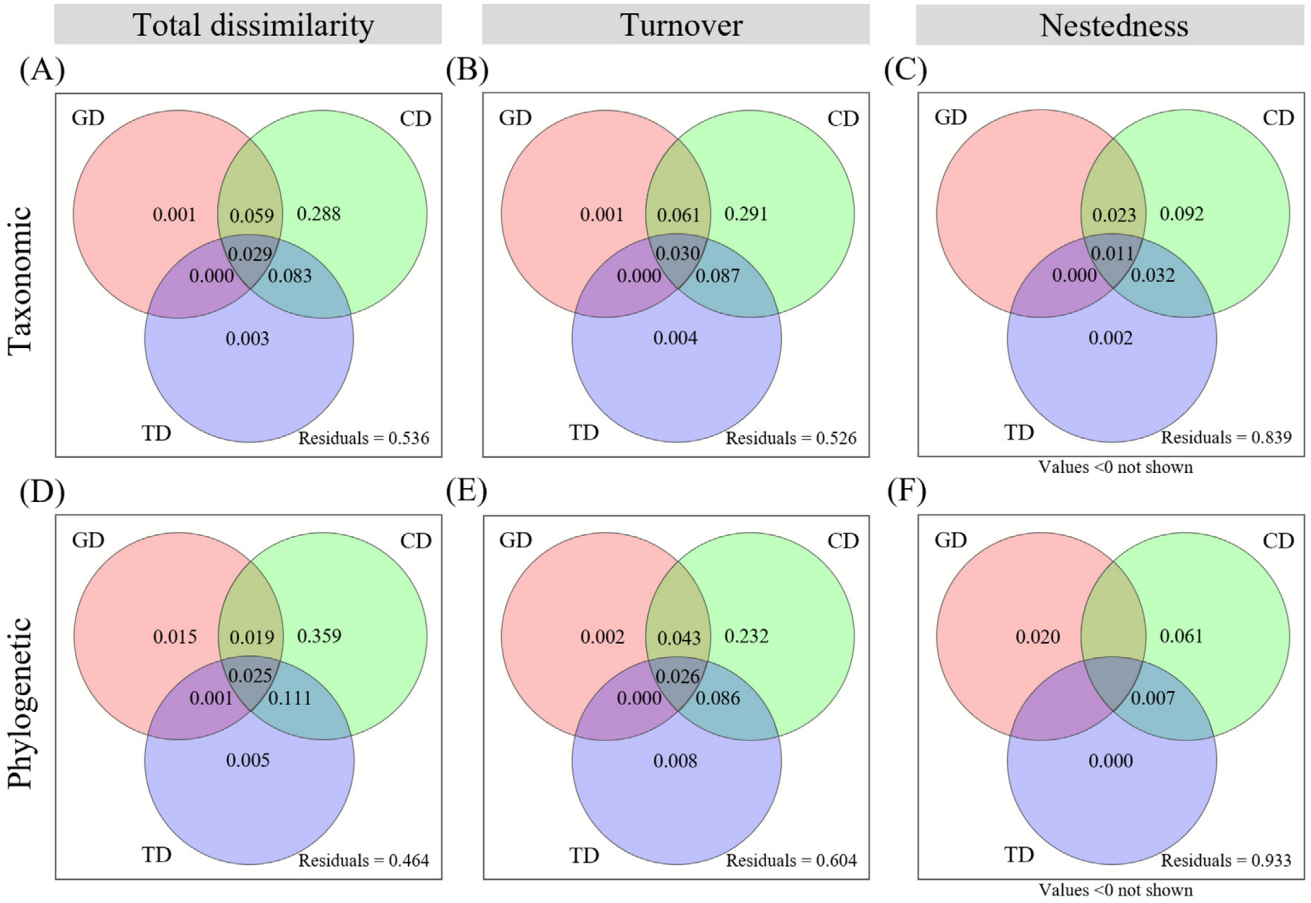
Variance partitioning of taxonomic (A–C) and phylogenetic (D–F) β‐diversity and their turnover and nestedness components in mountain plant communities. The unique and shared contributions of geographic distance (GD), climatic distance (CD), and topographic distance (TD) are shown, with negative values omitted (see Table S2). The proportions represent coefficients of determination (*R* ^2^) derived from multiple regression on distance matrices (MRM), where *R* ^2^ refers to the adjusted coefficient of determination (adjusted *R* ^2^). Climatic distance explains the largest independent fraction, reflecting the predominant influence of climatic heterogeneity on the spatial differentiation of plant communities.

Variance partitioning analysis (Figure 4) confirmed that climatic distance uniquely explained the largest fraction of β‐diversity, contributing 28.8% and 35.9% to total taxonomic and phylogenetic β‐diversity, respectively. For turnover, climatic distance accounted for 29.1% and 23.2%, while for nestedness the values were 9.2% and 6.1%. These results indicate that temperature and precipitation gradients play a dominant role in structuring plant communities, reflecting strong environmental filtering effects in this arid mountain system.

#### 3.3.3 Temperature as the Dominant Driver of Community Differentiation

Further analysis of individual environmental variables (Table 4) revealed that mean annual temperature (MAT) was the primary environmental determinant of both taxonomic and phylogenetic β‐diversity, underscoring the importance of thermal energy in shaping community composition along elevation. In contrast, slope and mean annual precipitation (MAP) showed weaker or more localized influences, while aspect and topographic wetness index (TWI) were not significant (*p* > 0.05). Collectively, these findings suggest that temperature‐driven environmental filtering, rather than terrain‐related microhabitat variation, predominates in controlling the spatial differentiation of plant communities in this extremely arid mountain landscape.

**TABLE 4:** Results of multiple regression on distance matrices (MRM) for taxonomic and phylogenetic β‐diversity (Tβsor, Pβsor), and their turnover (Tβsim, Pβsim) and nestedness (Tβnes, Pβnes) components, based on distance matrices of different climatic (MAT, MAP) and topographic (Slope, Aspect, TWI) variables.

Index	MAT	MAP	Slope	Aspect	TWI	*R* ^2^	Adjusted *R* ^2^	*p*
Tβsor	0.187	NS	NS	NS	NS	0.530	0.529	< 0.001
Tβsim	0.213	NS	NS	NS	NS	0.534	0.533	< 0.001
Tβnes	−0.027	−0.007	NS	NS	NS	0.179	0.177	< 0.001
Pβsor	0.168	NS	NS	NS	NS	0.599	0.598	< 0.001
Pβsim	0.125	NS	−0.023	NS	NS	0.435	0.434	< 0.001
Pβnes	0.043	−0.022	0.021	NS	NS	0.141	0.139	< 0.001

*Note:* *R* ^2^ indicates the proportion of variation in β‐diversity explained by all variables in the model, and Adjusted *R* ^2^ accounts for model complexity. Partial regression coefficients (b) and associated *p*‐values were obtained from permutation tests (9999 runs). Significance level is *p* < 0.05; NS indicates no significance. Ecologically, higher *R* ^2^ values indicate that climatic and topographic gradients exert stronger environmental filtering effects on plant community β‐diversity along the mountain gradient.

Abbreviations: MAP, mean annual precipitation; MAT, mean annual temperature; TWI, topographic wetness index.

## 4 Discussion

Species turnover was identified as the dominant component of both taxonomic and phylogenetic β‐diversity in the northern slope of the central Kunlun Mountains. This pattern is highly consistent with findings from regions such as the Qinghai–Tibet Plateau, the Helan Mountains, and the Inner Mongolia grasslands (Li et al. 2021, 2024; Zheng et al. 2022), and echoes the global paradigm of turnover‐dominated β‐diversity highlighted by Soininen et al. (2018). This turnover‐dominated pattern reflects strong environmental heterogeneity and spatial isolation of the region, where steep gradients in temperature and precipitation promote niche differentiation and species substitution across habitats. The relatively weaker phylogenetic turnover compared with the taxonomic dimension suggests that climatic oscillations since the Quaternary have primarily driven species replacement within closely related clades, leading to phylogenetic clustering shaped by both environmental filtering and limited post‐glacial dispersal (Cavender‐Bares et al. 2009; Du et al. 2022; Wen et al. 2024). Although relatively low species richness and the use of a synthesis‐based phylogeny may reduce the resolution and statistical power for detecting fine‐scale patterns of phylogenetic β‐diversity (e.g., due to polytomies) (Swenson 2009), such approaches are widely adopted and considered robust for comparative analyses across strong environmental gradients (Qian et al. 2020, 2024), and the main conclusions regarding turnover‐dominated assembly processes are unlikely to be affected.

The influence of elevational gradients further accentuates the dynamic nature of community composition in arid mountain environments. As elevation increases, pronounced changes in temperature, precipitation, and habitat structure promote rapid species and lineage turnover, thereby intensifying community differentiation (Yao et al. 2024). In addition to contemporary environmental heterogeneity, the high turnover along elevation may also reflect the legacy of historical refugia during Quaternary glacial cycles. The complex topography of the central Kunlun Mountains likely provided multiple microrefugia that buffered lineages from regional extinction, allowing long‐term in situ persistence and subsequent postglacial recolonization from different elevations (Du et al. 2022; Wen et al. 2024). Such processes have generated distinct phylogenetic assemblages across altitudinal zones, reinforcing compositional dissimilarity through both ecological filtering and historical isolation. Consequently, biodiversity patterns in this region are shaped not only by spatial differentiation under current climatic gradients but also by the deep‐time imprint of glacial–interglacial dynamics on lineage turnover.

Climatic distance, particularly mean annual temperature, emerged as the strongest environmental determinant of spatial β‐diversity. Temperature not only determines plant distribution limits, population viability, and phenological timing, but also exerts a profound influence on community succession trajectories and the rate of phylogenetic diversification (Wiens and Graham 2005; Donoghue 2008). The decomposition of β‐diversity further demonstrated that environmental variables accounted for substantially greater variation in the turnover component compared to nestedness, indicating that community differentiation in this region is primarily governed by environmental filtering, while nestedness is more closely related to local species loss or extreme microhabitat conditions (Yao et al. 2024). Building on this, climatic heterogeneity was found to enhance niche differentiation and species adaptation, thereby further facilitating dynamic turnover among species and lineages (Graham and Fine 2008; Svenning et al. 2015). Thus, temperature emerges as a keystone factor in maintaining biodiversity in these extremely arid mountain ecosystems (Hawkins et al. 2003; Chen et al. 2025). Nevertheless, approximately half of the total variation in β‐diversity remained unexplained, highlighting the likely influence of unmeasured variables such as soil properties, microclimatic variability, and land‐use or anthropogenic disturbances, which warrant further investigation.

Theoretically, this mechanism aligns with the classic “water‐energy dynamics hypothesis” for arid environments, emphasizing that energy supply (e.g., temperature) plays a dominant role in shaping community structure where water is limiting (O'Brien 1998). Although geographic and topographic factors do contribute to community differentiation, and dispersal barriers restrict the migration and exchange of some species and lineages, it is strong environmental filtering at macroecological scales and under extreme climatic conditions that predominates (Qian and Ricklefs 2007; He et al. 2022). Nevertheless, limited dispersal may still play a non‐negligible role at finer spatial scales, particularly within isolated valleys and slope mosaics where seed dispersal is constrained by rugged terrain and sparse vegetation cover. Such localized dispersal limitation could reinforce species turnover by restricting colonization opportunities, thereby interacting with environmental filtering to jointly shape β‐diversity patterns in this arid mountain landscape (Svenning et al. 2015; Yao et al. 2024).

As a comparison across ecosystem types, Zhou et al. (2023) found that the β‐diversity of aquatic angiosperms in China was mainly structured by climatic and geographic distances, but the high hydrological connectivity of aquatic habitats led to lower species turnover and stronger nestedness. In contrast, the arid mountain ecosystems of the Kunlun region exhibit steep environmental gradients and limited connectivity, where strong environmental filtering and dispersal limitation jointly promote pronounced species and lineage replacement. This contrast highlights how habitat connectivity and climatic harshness together determine the dominant mechanisms driving β‐diversity across terrestrial and aquatic ecosystems.

From a conservation ecology perspective, the compositional features of β‐diversity directly inform strategies for the spatial design of protected areas. Previous research suggests that when β‐diversity is dominated by nestedness, a single large reserve is preferable; in contrast, when turnover is dominant, a network of several small reserves is more effective for maximizing overall diversity (Bergamin et al. 2017; Arif et al. 2022). In this study, given the turnover‐dominated β‐diversity patterns observed in both taxonomic and phylogenetic dimensions, we recommend prioritizing a spatially distributed reserve network on the northern slope of the central Kunlun Mountains to better conserve the region's unique species and phylogenetic diversity. However, the extreme remoteness, harsh climate, and limited accessibility of this region may constrain large‐scale management implementation. Therefore, conservation planning should balance biodiversity representation with practical feasibility by integrating smaller, ecologically representative conservation units into existing management frameworks—such as local grassland restoration zones or national ecological barrier programs—to optimize resource allocation and ensure long‐term ecosystem resilience.

In summary, the spatial patterns of β‐diversity in arid mountain plant communities are primarily shaped by climatic gradients, with temperature as the dominant driver, while geographic and topographic factors play secondary roles. Our study deepens understanding of biodiversity assembly mechanisms under extreme environmental stress and provides a robust scientific basis for conservation and management in ecologically fragile arid regions. Future research should further integrate soil variables, plant functional traits, and long‐term, multi‐scale monitoring to comprehensively elucidate the diversity and complexity of community assembly and adaptation mechanisms under extreme environments, which, in the context of climate change, will thereby facilitate a more comprehensive insight into the dynamic processes and adaptive potential of arid mountain ecosystems.

## 5 Conclusion

This study systematically elucidated the patterns and driving mechanisms of β‐diversity in mountain plant communities on the northern slope of the central Kunlun Mountains. The results indicate that, in both taxonomic and phylogenetic dimensions, β‐diversity is predominantly driven by species turnover, with overall β‐diversity and its components significantly increasing with elevational differences. Climatic distance, especially mean annual temperature, emerged as the key factor influencing the spatial variation of regional β‐diversity. This research provides a theoretical basis for formulating biodiversity conservation and ecological management strategies under climate change and suggests that establishing multiple small nature reserves would be more effective for conserving the unique species and phylogenetic diversity of this region.

## Author Contributions

**Tiantian Qin:** conceptualization (equal), data curation (lead), formal analysis (lead), investigation (lead), methodology (lead), visualization (equal), writing – original draft (lead). **Hongyang Chen:** conceptualization (equal), data curation (equal), investigation (equal), methodology (equal), software (supporting). **Shengjie Chen:** conceptualization (supporting), data curation (equal), investigation (equal), methodology (equal). **Pengwei Zhang:** conceptualization (supporting), investigation (equal), methodology (equal), resources (equal). **Zhifang Xue:** conceptualization (equal), investigation (equal), software (equal). **Shengtianzi Dong:** conceptualization (equal), funding acquisition (equal), supervision (equal), writing – review and editing (equal). **Hanyue Wang:** conceptualization (lead), funding acquisition (lead), methodology (equal), supervision (lead), writing – review and editing (lead).

## Funding

This work was supported equally by Tianchi Talent Project of Xinjiang (Grant CZ001622); High‐level Talents Scientific Startup Project of Shihezi University (Grant RCZK202472); Water Resources and Aquatic Ecology Investigation Project in the Hotan River Basin of the Tarim Basin (Grant DD20220962).

## Conflicts of Interest

The authors declare no conflicts of interest.

## Supporting information


**Table S1:** ece372886‐sup‐0001‐TableS1.pdf.

**Table S2:** ece372886‐sup‐0002‐TableS2.pdf.

## Data Availability

All data and codes used to support and analyses the conclusions of this study are available at https://doi.org/10.5281/zenodo.16568146.
