# Supplementary material for: Patterns and Driving Mechanisms of β‐Diversity in Mountain Plant Communities of Arid Regions
Source: Ecol Evol. 2026 Jan 12;16(1):e72886. doi: 10.1002/ece3.72886 (PMC12795618; doi:10.1002/ece3.72886)
Supplement: Supplementary file 1 — Table S1: ece372886‐sup‐0001‐TableS1.pdf. [file ECE3-16-e72886-s001.pdf]

TABLE S1 | The list of species

FAMILY	GENUS	SPECIES
Poaceae	*Achnatherum*	*Achnatherum splendens*
Poaceae	*Aristida*	*Aristida adscensionis*
Poaceae	*Elymus*	*Elymus nutans*
Poaceae	*Elymus*	*Elymus sibiricus*
Poaceae	*Elytrigia*	*Elytrigia repens*
Poaceae	*Eragrostis*	*Eragrostis minor*
Poaceae	*Festuca*	*Festuca brachyphylla*
Poaceae	*Festuca*	*Festuca ktyloviana*
Poaceae	*Festuca*	*Festuca olgae*
Poaceae	*Koeleria*	*Koeleria litvinowii*
Poaceae	*Leymus*	*Leymus secalinus*
Poaceae	*Poa*	*Poa relaxa*
Poaceae	*Schismus*	*Schismus arabicus*
Poaceae	*Stipa*	*Stipa breviflora*
Poaceae	*Stipa*	*Stipa robarowskyi*
Poaceae	*Stipa*	*Stipa glareosa*
Poaceae	*Stipa*	*Stipa purpurea*
Poaceae	*Stipa*	*Stipa subsessiliflora*
Asteraceae	*Ajania*	*Ajania fruticulosa*
Asteraceae	*Artemisia*	*Artemisia sieversiana*
Asteraceae	*Artemisia*	*Artemisia mongoliea*
Asteraceae	*Artemisia*	*Artemisia xerophytica*
Asteraceae	*Artemisia*	*Artemisia demissa*
Asteraceae	*Aster*	*Aster alpinus*
Asteraceae	*Cirsium*	*Cirsium lanatum*
Asteraceae	*Echinops*	*Echinops gmelini*
Asteraceae	*Hexinia*	*Hexinia polydichotoma*
Asteraceae	*Leontopodium*	*Leontopodium nanum*
Asteraceae	*Leontopodium*	*Leontopodium ochroleucum*
Asteraceae	*Mulgedium*	*Mulgedium tataricum*
Asteraceae	*Seriphidium*	*Seriphidium rhodanthum*
Asteraceae	*Taraxacum*	*Taraxacum mongolicum*
Fabaceae	*Astragalus*	*Astragalus tibetanus*
Fabaceae	*Astragalus*	*Astragalus nematodioides*
Fabaceae	*Astragalus*	*Astragalus densiflorus*
Fabaceae	*Caragana*	*Caragana polourensis*
Fabaceae	*Oxytropis*	*Oxytropis biloba*
Fabaceae	*Oxytropis*	*Oxytropis kansuensis*
Fabaceae	*Oxytropis*	*Oxytropis ochrocephala*
Fabaceae	*Oxytropis*	*Oxytropis falcata*
Fabaceae	*Oxytropis*	*Oxytropis densa*
Fabaceae	*Oxytropis*	*Oxytropis puberula*

TABLE S1 | (Continued)

FAMILY	GENUS	SPECIES
Fabaceae	*Oxytropis*	*Oxytropis glabra*
Fabaceae	*Oxytropis*	*Oxytropis microphylla*
Fabaceae	*Sphaerophysa*	*Sphaerophysa salsula*
Amaranthaceae	*Agriophyllum*	*Agriophyllum squarrosum*
Amaranthaceae	*Atriplex*	*Atriplex centralasiatica*
Amaranthaceae	*Bassia*	*Bassia dasyphylla*
Amaranthaceae	*Ceratoides*	*Ceratoides latens*
Amaranthaceae	*Corispermum*	*Corispermum mongolicum*
Amaranthaceae	*Salsola*	*Salsola collina*
Amaranthaceae	*Sympegma*	*Sympegma regelii*
Gentianaceae	*Comastoma*	*Comastoma falcatum*
Gentianaceae	*Comastoma*	*Comastoma pedunculatum*
Gentianaceae	*Gentiana*	*Gentiana pseudoaquatica*
Gentianaceae	*Gentiana*	*Gentiana karelinii*
Gentianaceae	*Gentianella*	*Gentianella pygmaea*
Gentianaceae	*Gentianopsis*	*Gentianopsis barbata*
Gentianaceae	*Lomatogonium*	*Lomatogonium carinthiacum*
Brassicaceae	*Capsella*	*Capsella bursapastoris*
Brassicaceae	*Draba*	*Draba altaica*
Brassicaceae	*Draba*	*Draba oreades*
Brassicaceae	*Lepidium*	*Lepidium apetalum*
Brassicaceae	*Torularia*	*Torularia humilis*
Amaryllidaceae	*Allium*	*Allium kaschianum*
Amaryllidaceae	*Allium*	*Allium przewalskianum*
Amaryllidaceae	*Allium*	*Allium caricoides*
Cyperaceae	*Carex*	*Carex atrofusca*
Cyperaceae	*Kobresia*	*Kobresia humilis*
Orobanchaceae	*Cistanche*	*Cistanche deserticola*
Orobanchaceae	*Pedicularis*	*Pedicularis cheilanthifolia*
Plantaginaceae	*Plantago*	*Plantago depressa*
Plantaginaceae	*Plantago*	*Plantago minuta*
Primulaceae	*Androsace*	*Androsace squarrosula*
Primulaceae	*Androsace*	*Androsace flavescens*
Ranunculaceae	*Clematis*	*Clematis tangutica*
Ranunculaceae	*Pulsatilla*	*Pulsatilla campanella*
Rosaceae	*Potentilla*	*Potentilla multifida*
Rosaceae	*Potentilla*	*Potentilla bifurca*
Apiaceae	*Ligusticum*	*Ligusticum mucronatum*
Asparagaceae	*Asparagus*	*Asparagus persicus*
Boraginaceae	*Lappula*	*Lappula stricta*
Campanulaceae	*Adenophora*	*Adenophora himalayana*
Caryophyllaceae	*Silene*	*Silene songarica*

TABLE S1 | (Continued)

FAMILY	GENUS	SPECIES
Celastraceae	*Parnassia*	*Parnassia laxmanni*
Crassulaceae	*Rhodiola*	*Rhodiola pamiroalaica*
Geraniaceae	*Geranium*	*Geranium collinum*
Iridaceae	*Iris*	*Iris loczyi*
Papaveraceae	*Hypecoum*	*Hypecoum leptocarpum*
Polygonaceae	*Polygonum*	*Polygonum viviparum*
Violaceae	*Viola*	*Viola kunawarensis*
