# Supplementary material for: Patterns and Driving Mechanisms of β‐Diversity in Mountain Plant Communities of Arid Regions
Source: Ecol Evol. 2026 Jan 12;16(1):e72886. doi: 10.1002/ece3.72886 (PMC12795618; doi:10.1002/ece3.72886)
Supplement: Supplementary file 2 — Table S2: ece372886‐sup‐0002‐TableS2.pdf. [file ECE3-16-e72886-s002.pdf]

TABLE S2 | Variance partitioning analysis (VPA) results of taxonomic and phylogenetic β-diversity and their components.

Index	GD (unique)	CD (unique)	TD (unique)	GD&CD (shared)	GD&TD (shared)	CD&TD (shared)	GD&CD&TD (shared)	Residuals	Total Adj.R^2
Tβsor	0.00139	0.28804	0.00294	0.05886	0.08307	0.00025	0.0291	0.53633	0.46367
Tβsim	0.00122	0.29128	0.00354	0.0611	0.08652	0.00026	0.03028	0.52579	0.47421
Tβnes	-0.00016	0.09231	0.00226	0.02253	0.03247	0.00008	0.0114	0.83912	0.16088
Pβsor	0.01493	0.35916	0.00541	0.01928	0.11071	0.00104	0.02533	0.46415	0.53585
Pβsim	0.00172	0.2315	0.00789	0.04254	0.08591	0.00046	0.02605	0.60393	0.39607
Pβnes	0.01957	0.06058	0	-0.01925	0.00701	-0.00024	-0.00032	0.93265	0.06735

Note: GD (unique), CD (unique), and TD (unique) represent the unique explanatory fractions of geographical distance (GD), climatic distance (CD), and topographic distance (TD), respectively. GD&CD (shared), GD&TD (shared), and CD&TD (shared) denote the shared explanatory fractions between any two environmental factors (excluding the third factor). GD&CD&TD (shared) represents the shared fraction explained jointly by all three factors. Residuals indicate the unexplained fraction. Total Adj.R^2 refers to the total adjusted coefficient of determination (Adjusted R^2) of the three-factor model. Negative values suggest weak explanatory power or overlapping errors possibly caused by collinearity among variables. All fractions represent adjusted R^2 values derived from variance partitioning analysis (VPA).
